# Global Immunization Crisis Amid the COVID-19 Pandemic: Implications for Pediatric Oncology

**DOI:** 10.1200/GO.23.00477

**Published:** 2024-02-29

**Authors:** Syed Ibrahim Bukhari, Fyezah Jehan, Asim Belgaumi

**Affiliations:** ^1^ Department of Oncology, Aga Khan University, Karachi, Pakistan; ^2^ Department of Pediatrics and Child Health, Aga Khan University, Karachi, Pakistan

## Abstract

Declining herd immunity and severe manifestation of vaccine preventable infections underscores the need for vaccinations campaigns to urgently vaccinate children who missed their routine immunizations.

The global impact of the COVID-19 pandemic caused by SARS-CoV-2 infection is staggering; as of December 11, 2023, there were over 77.2 million reported cases and 6.98 million deaths.^1^ Beyond the immediate health crisis, the pandemic has cast a long and ominous shadow, challenging health care systems worldwide. At its peak, health care capacities were strained in most countries, presenting unprecedented challenges for health care workers and hospital staff who found themselves grappling with depleting basic care necessities for both their patients and themselves. This upheaval extended to routine patient care, disrupting critical services like well-child care and treatment of other illnesses. A postpandemic study has projected that the disruptions in cancer care during the COVID-19 pandemic could potentially result in 21,247 (2.0%) more cancer deaths in Canada from 2020 to 2030, translating to a staggering 355,172 life years lost because of pandemic-related diagnostic and treatment delays.^2^ Additional studies from across the world have predicted a similar negative impact of treatment delays and omissions on adult and childhood cancer outcomes.^3-6^

In addition to the catastrophic consequences of prolonged lockdowns, one of the indirect yet profound effects of the COVID-19 pandemic has been the disruption of routine community immunization programs. This was identified and reported as a potential collateral threat early during the pandemic^7,8^ and has subsequently been verified as a public health emergency.^9-13^ Most vaccine-preventable illnesses require a particular proportion of the population to be immune to stop the chain of transmission and achieve herd immunity. For highly contagious diseases like measles, for example, over 95% of the population needs to be immune to effectively stop sustained disease transmission and achieve herd immunity.^14^ During the COVID-19 pandemic, disruptions in routine vaccination programs have raised concerns about declining immunity levels in the population against various diseases. Of note, 73 countries had a backslide in vaccination rates during the pandemic, with 15 having bounced back and 24 making progress toward recovery. However, worryingly, 34 low- and middle-income countries (LMIC) are either stalled or still facing a decline in vaccination coverage.^15^ This disruption is a result of various factors, including the pandemic-related lockdowns, diversions of health care resources, and the subsequent economic downturn, resulting in shifting national health priorities. The missed vaccinations at the community level have led to a loss of community-level immunity globally, particularly affecting LMIC. Because of disease- and treatment-induced loss of humoral immunity, children with cancer rely on herd immunity to protect themselves from common infections.

Pediatric oncology patients, already navigating the complexities of weakened immune systems, find themselves at an elevated risk of infections from a spectrum of microorganisms, both typical and atypical.^16,17^ Of particular concern are viral infections, which often present unusual symptoms in patients with impaired immune systems and have considerably worse outcomes.^18^ Common diseases of childhood, like chicken pox, incur a serious morbidity and mortality risk for children undergoing cancer therapy.^19^ CNS manifestations of viral infections, such as varicella zoster, herpes simplex and measles, can be protean and overlapping, making clinical diagnoses difficult.^20-22^ Measles infections are particularly concerning because of its diverse CNS manifestations in immunocompromised hosts, such as acute measles encephalitis, subacute measles inclusion-body encephalitis, epilepsia partialis continua, and subacute sclerotic panencephalitis.^23-28^ This variability in presentation and rarity of its occurrence complicates timely consideration and identification. These are often not part of our usual list of differential diagnoses.

In the article that accompanies this editorial, Sharma et al^29^ presented findings from an analysis of 18 children with leukemia, each exhibiting symptoms of ataxia, myoclonic jerks, and focal seizures. Although this outbreak was eventually identified as measles inclusion-body encephalitis, only four of these children had a documented history of the typical measles-like rash. Even after the administration of ribavirin, antiepileptic medications, intravenous immunoglobulin, and in some cases, methylprednisolone, most patients ultimately required intubation with ventilatory support because of intractable symptoms. Only five of the 18 children survived, albeit with significant residual neurologic deficits.

The identification of the clustering of patients with these symptoms and the rigorous investigation into the causative factors conducted by Sharma et al^29^ are commendable. A detailed epidemiologic outbreak investigation was performed, with focused serologic and molecular confirmation of the measles infection once it was suspected. Their ability to think outside the conventional diagnostic framework for uncommon presentations of common infectious illnesses is particularly noteworthy. The paper underscores the significant morbidity and mortality associated with atypical manifestations of measles in pediatric patients with cancer and highlights the need for oncologists, adult and pediatric, to consider infectious illnesses that are not routinely encountered as a potential etiology when patients present with uncommon and challenging symptoms. This is particularly likely to happen with infections that are part of the routine immunization schedule for children, such as measles, mumps, or polio. Because of vaccine effectiveness, most physicians are less aware of common and uncommon presenting signs and symptoms and may miss timely diagnosis, leading to potential serious adverse outcomes, including continuous exposure to other patients and health care providers.

The double jeopardy situation of declining herd immunity and atypically manifested vaccine preventable infections in pediatric patients with cancer underscores the need for focused strategies to mitigate these impacts and protect these vulnerable patients. Initiating campaigns for catch-up vaccinations to urgently vaccinate children who missed their routine immunizations is a health care imperative and a moral obligation. It is particularly crucial to tailor these campaigns to reach children after the age they would normally be vaccinated, ensuring that they receive necessary doses. These need to go hand in hand with other vaccine improvement measures including reaching children who have never received a vaccine, strengthening vaccine confidence, prioritizing funding for primary health care, and restoring health workforces and infrastructure. Only then will we be able to restore herd immunity and offer quality cancer care, thus averting the collateral damage inflicted by the pandemic on pediatric oncology and ensuring a brighter, healthier future for these resilient young patients.
